# Mars sample return campaign: biological risk and a proposed sample safety assessment protocol

**DOI:** 10.1128/aem.02461-25

**Published:** 2026-05-27

**Authors:** G. McDonnell, B. L. Teece, R. Mackelprang, N. Cressie, J. R. McQuiston, L. E. Mayhew, A. Steele, S. Siljeström, B. M. Ahern, N. Baird, R. E. Davis, K. L. French, M. Glamoclija, H. Graham, K. B. Hummel, W. Page, N. Pearce, A. Regberg, D. Relman, M. A. Sephton, B. Sherwood Lollar, J. Vanhomwegen, M. B. Wilhelm, B. Shirey, D. W. Beaty

**Affiliations:** 1 Johnson & Johnson 30212 , Raritan, New Jersey, USA; 2 Jet Propulsion Laboratory, California Institute of Technology 6469, Pasadena, California, USA; 3 Cornell Center for Astrophysics and Planetary Science, Department of Astronomy, Cornell University 5922, Ithaca, New York, USA; 4 California State University 14671, Northridge, California, USA; 5 University of Wollongong 8691, Wollongong, New South Wales, Australia; 6 Centers for Disease Control and Prevention 1242, Atlanta, Georgia, USA; 7 University of Colorado 1877, Boulder, Colorado, USA; 8 Carnegie Institute for Science 1485, Washington, DC, USA; 9 Rise Institutes of Sweden 388792, Gothenburg, Sweden; 10 Department of Defense 1276 , Washington, DC, USA; 11 Texas State University 7174, San Marcos, Texas, USA; 12 NASA Johnson Space Center 43834 , Houston, Texas, USA; 13 U.S. Geological Survey, Reston, Virginia, USA; 14 Rutgers University 242612, New Brunswick, New Jersey, USA; 15 NASA Goddard Space Flight Center 53523, Greenbelt, Maryland, USA; 16 London School of Hygiene 4906, London, United Kingdom; 17 Stanford University 6429, Stanford, California, USA; 18 Imperial College 4615, London, United Kingdom; 19 University of Toronto 7938, Toronto, Canada; 20 Institut Pasteur 27058, Paris, France; 21 NASA AMES Research Center 53406 , Moffett Field, California, USA; Michigan State University, East Lansing, Michigan, USA

**Keywords:** space travel, biohazard, life detection, astrobiology

## Abstract

Returning surface samples from Mars to Earth has been a major planetary science objective, with the potential for the detection of microbiological life and the possibility of improving our understanding of the origins of life. The National Aeronautics and Space Administration and the European Space Agency assembled a team to assess the level of risk that returned samples could contain potential biohazards. The team was chartered with optimizing previous sample safety assessment strategies, defining what constitutes a biological hazard, developing a protocol to test for biohazards, and establishing a statistical framework to determine if samples may be safe for release from a high-containment facility. This report presents the biological context for a proposed three-step protocol for testing returned samples, including how to determine if microorganisms are present, and if they could be (or were recently) alive.

## THREE-STEP PROTOCOL

**Fig 1:**
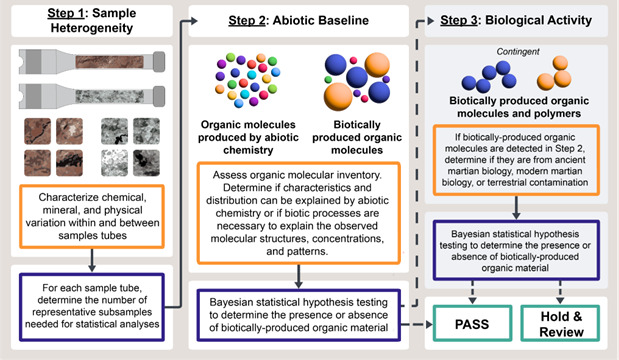
Overview of the proposed sample safety assessment protocol summarizing the workflow and decision-making processes. Reprinted from reference 5, previously published under a CC BY 4.0 license.

The National Aeronautics and Space Administration (NASA)/European Space Agency (ESA) Sample Safety Assessment Protocol Tiger Team (SSAP-TT) included a multi-disciplinary and international team of experts. They consisted of co-chairs, facilitators, science members, and ex officio members across government agencies, academia, and private industry. During September 2023–August 2024, they developed a proposed three-step protocol, supported by a Bayesian statistical framework, to assess the level of risk of returned samples from the Mars surface containing biohazards that could harm the Earth’s biosphere. This protocol would be necessary for planetary protection, preventing backward contamination of Earth (1). The protocol builds upon previous resources, including safety assessment strategies (2), astrobiology strategy (3), and the 2023 Planetary Science Decadal Survey (4). The proposed protocol includes (Fig. 1) the following:

1. Understand the physical and chemical variation within and between the Mars sample tubes (i.e., sample heterogeneity). Investigate to determine the number of representative subsamples needed to be analyzed using Bayesian statistical methods.
2. Comparing samples to the abiotic baseline. Assess the organic molecular inventory of subsamples in each sample tube against the abiotic background. Assess if any organic molecules can be explained by abiotic chemistry or by biotic processes (5).
3. Assess risks of biological activity. This assessment would be a more detailed analysis of samples, followed by additional measurements as needed to determine the source of the signal. This considers (i) whether a biological signal is ancient/fossilized or modern and recently active and, (ii) if modern, whether it originates from Martian biology or terrestrial contamination.

A Bayesian statistics approach was developed to evaluate the cumulative data from analysis (N. Cressie, unpublished data). This allows for limited sample consumption and cumulative biohazard risk assessment, including prior knowledge. The protocol was designed to accommodate new data inputs, technology advancements, and updated assessments of risk tolerance to give confidence in the final assessment.

## MARS SAMPLE COLLECTION

As an example, the Mars 2020 Perseverance Rover is collecting surface samples inside and on the periphery of Jezero Crater on Mars (6–8). This comprises a collection of igneous and sedimentary samples of approximately 10–25 g each (9–11). The crater is a dry, wind-swept desert that includes sand dunes, mountains, ridges, blocky erosion products, unconsolidated rock, and dust (“regolith”). No liquid water or ice has been detected, and the water content in the atmosphere is extremely low (11). The surface does not support conditions consistent with known habitability and is unlikely to contain extant life, as the surface is arid, oxidizing, cold, and subject to high radiation doses (11–15).

Scientific interest in these samples includes the hypothesis that Mars could have hosted life in its ancient past. Ancient fossilized life is not considered hazardous to the Earth’s biosphere, given the high likelihood that it would be biologically inactive and unable to reproduce. Although the risk is low, it is not zero. The return of samples to Earth would propose initial handling and analysis in a biologically secure high-containment sample receiving facility. The proposed protocol is an extension of previous work (2, 16), and is currently under consideration by NASA/ESA.

## BIOLOGICAL CONTEXT

The agreed definition of “biological hazard” was a “biologically active Martian agent of organic origin capable of causing substantial harm to any component of the Earth’s biosphere if allowed out of containment.” These agents could include opportunistic pathogenic microorganisms or bioreactive substances that may multiply. Hazards to an Earth-based ecosystem could include an invasive species that eliminates a terrestrial organism by niche competition. This definition aligns with NASA’s accepted definition for “bioactive molecules” when identifying biological potential planetary protection hazards (17). Since the Outer Space Treaty of 1967, NASA’s Office of Planetary Protection and others have worked to set and maintain recommended controls to prevent the contamination of other planetary bodies from Earth microbes (“forward planetary protection”) and avoid exposing Earth to extraterrestrial life or bioactive molecules (“backward planetary protection” [18]). In accordance with the precautionary principle, the future return of Mars material should be assessed to protect the Earth from harm (1, 11, 13–15).

The ability of detectable material to be biologically active or to multiply could cause potential harm to Earth’s biosphere (2, 18). No single practical microbiological (or molecular) test or even series of tests could be proposed to rule out the presence of extraterrestrial microbial life, as exemplified by our knowledge of the wide spectrum of life in Earth’s biosphere. Therefore, a framework was recommended centered on the idea that the detection and analysis of organic molecules that are indicative of biological activity, along with textural and mineralogical information, could be sufficient to determine the potential presence of a biological hazard.

Other definitions within the scope of this report included

- Biosignature: an object, substance, and/or pattern whose origin specifically requires a biological agent (4).
- Harm: the presence of a biological hazard outside of containment not associated with Earth contamination.

The detection of biosignatures in a sample that are not associated with potential contamination from Earth was determined to be sufficient to represent a potential hazard to Earth. The extent of potential hazard was not defined but the recommendation was made to contain such material under biosafety conditions for further assessment.

## COMPARING SAMPLES TO THE ABIOTIC BASELINE

Samples would first undergo a battery of tests to assess if abiotic or biotic processes contributed to the chemical inventory of the samples (5). Briefly, Earth life uses a relatively small contingent of monomers that polymerize into larger structures, such as membranes, proteins, and nucleic acids. To effectively search for life beyond Earth, astrobiologists and planetary scientists utilize the abiotic baseline, or “non-life background,” that defines the physicochemical parameters in potentially habitable environments in the absence of life processes (3, 4, 19). Since life’s signatures are typically concentrated in a distribution that is less diverse (or different) from abiotic processes, it is possible to distinguish the presence of biotic from abiotic chemistry. An advantage of this approach is that it makes no assumptions about the nature of potential Martian life other than that it could be carbon based and could utilize a distinct chemical alphabet. There has been a revolution in the ability to interrogate and understand microbial communities in their environmental context. Most microbial life cannot be readily grown in laboratories, so notable advances have been made in understanding the biology that blurs the line between living and non-living (e.g., viruses and prions). Advances include detecting life in low-biomass terrestrial analogs, understanding the environmental context in which it occurs, and determining how life alters its environment.

The proposed abiotic baseline assessment (Step 2) would be based on preexisting knowledge of the Martian environment; chemical, mineral, and physical heterogeneity within and between samples; and the analysis, characterization, and distribution of potential organic molecules (5), and then these data would be fed into a Bayesian statistical model (N. A. Cressie, unpublished data). The microbiological assessment (Step 3) begins only if there is evidence of life in the sample. The team agreed that a “hold and review” must take place to determine if the signal was capable of reproducing and causing harm (2).

## EVIDENCE OF BIOLOGICAL ACTIVITY

**Fig 2:**
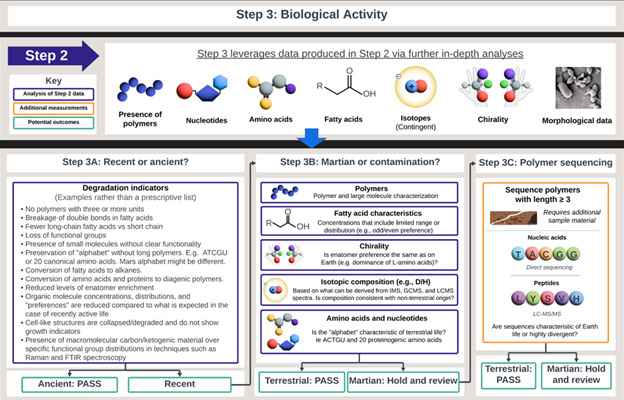
Summary of the proposed Step 3 (biological activity).

The SSAP proposed (i) to determine if the biotic signal originated from ancient life when the Martian surface was potentially more habitable or if it is from extant (recent) life and (ii) to understand if the signal originates from extant Martian biology or from terrestrial contamination. This assessment would utilize data collected from contamination monitoring (20, 21) in the previous steps, but more detailed or repeated analysis of samples may be necessary (Fig. 2). This was designed to minimize sample extraction and the amount of the core samples, while maximizing biological risk hazard assessment.

A key property of life is the ability to replicate. If data consistent with extant and active biology (including recent life) that are not definitively from Earth are observed, this may lead to the conclusion that something in the sample has the potential to replicate and be hazardous. The inverse is also true; however, assessing replicative ability is not initially experimentally feasible or sufficiently reliable. In terrestrial environments such as soils, it is well known that only a subset of viable microbes can be grown in the laboratory, depending on growth conditions. For putative Martian biology, understanding or triggering an unknown life form’s reproductive mechanism is likely to be even more difficult. Although it is possible to determine experimentally whether the samples may contain signs of life, the team was not able to identify at this time definitive ways to determine if Mars-sourced biochemistry may be alive and capable of reproduction.

## ANCIENT VS EXTANT LIFE

Step 3 would re-analyze data from Step 2 to provide information about the distribution of functionalized vs diagenetically altered organic compounds to yield clues as to the metabolic state of the organic material (e.g., live/dead/degraded). As organisms die and decay, the organic molecules associated with them undergo a degradation process that alters the original molecular inventory. On Earth, organic material sourced from ancient life is known to be highly distinct from modern life due to well-known alteration pathways, such as de-functionalization (22, 23). However, not all terrestrial alteration pathways may be relevant due to the unique environment of the Martian samples. For example, proteins and nucleic acids decompose geologically rapidly under the oxidizing and high-radiation levels observed in the Martian regolith (24, 25). The presence of individual alkanes, aromatic hydrocarbons, and peptides in the samples could indicate an original biotic origin resulting from the degradation of organic structures under environmental conditions and may represent a potential sign of extinct Martian life (22, 25). If results show that a sample contains evidence of ancient life but no evidence of viable life, the samples could be deemed to be non-hazardous (within a statistical confidence).

Analysis of these data depends on the types of biological polymers that may be identified, such as polymerized lipids, amino acids, nucleic acids, or other complex macromolecules. Examples of data that may distinguish ancient and recent biological sources include no polymers of three or more units, loss of functional groups (e.g., conversion of lipids to alkanes), presence of small or potentially fragmented molecules, conversion of peptides and proteins to diagenetic polymers, or the presence of macromolecular carbon/kerogenic material over specific functional group distributions (22, 25).

When considering the nature of organic signals, several principles are recommended. The highest-fidelity organic records should be sought so that source information can be identified, as organic records tend to lose fidelity during degradation. The diagnostic power of organic records decreases with a greater influence of the physical degradative environment that overprints the original biosynthetic process. It was also recognized that the susceptibility of different organic structures to degradation varies greatly (25). Agents of degradation include heat (26, 27), oxidation (28), irradiation (29), pressure (30), and biological activity (25). Ultimately, this consideration involves determining whether the detected structures (partially degraded or not) could have been generated by anything other than biological synthesis and whether the fidelity of the biomolecules is sufficient to indicate a viable organism. The ability to answer such technical questions will continue to improve.

## ASSESSMENT OF CONTAMINATION

The next task is to assess whether the signal is Martian in origin or from potential contamination by Earth life. Contamination knowledge during the various stages of the mission preparation, sampling, return, and laboratory analysis is important (20, 21). This includes the use of witness plates, controls, and process monitoring to characterize the types of contamination that may be present from terrestrial sources in the samples, quantitatively and qualitatively (31, 32). Mars samples are expected to contain low concentrations of organic carbon (<10–40 ppm), based on previous and current measurements (33–38). Such samples would be intrinsically vulnerable to terrestrial contamination. Sources could be from surfaces on any aspect of the flight hardware by biological or organic molecules (39). This could be monitored by sampling and strict controls throughout assembly and launch (20). Although sample tubes can be cleaned and sealed during spacecraft assembly to the required molecular and cleanliness levels to protect from contamination (40), it is not impossible that Earth-based microorganisms or remnants of these microorganisms could be present. Additionally, sources of organic compounds used on rover construction itself (e.g., trace contamination with lubricants) could have off-gassed or degraded and migrated into the sample tubes. A further source is after return, being contamination from the environment during sample handling on Earth despite strict containment practices. Once Martian samples are exposed to Earth’s environment, there is an immediate risk of extrinsic contamination even at a relatively low level. The use of controls during sample processing would be important to determine the background microbial and molecular contamination risks (21).

Negative and positive controls are referred to as contamination knowledge samples (32). These and contamination control are essential for all sample-based life detection missions/experiments. For example, a genetic inventory can be collected from the outbound sample-collecting equipment prior to launch since these contaminants have the potential to make the round trip and could be confused with any potential Martian signal (20, 40–42). This can include microbial characterization of the spacecraft assembly in cleanrooms. If one of these specific microorganisms or associated signatures was detected in a returned sample, it would immediately raise suspicion of contamination. The analysis of these controls can include fatty acid characteristics, chirality, isotopic characteristics, the presence of refractory carbon over more functional group distributions, and the alphabetic characteristics of any detected polymeric nucleotides and amino acids.

The consequences of misidentifying terrestrial contamination as evidence of life on Mars are significant. Therefore, ruling out terrestrial contamination to the maximum extent possible was identified as one of the top priorities. Two additional measurements were defined to further assess contamination, direct polymer sequencing of nucleic acids and peptides.

## DIRECT POLYMER SEQUENCING

Theoretically, the cumulative analysis defined above may be sufficient to determine that polymers and large molecules present are highly likely to be sourced from terrestrial or Martian sources (within a statistical confidence). If this is not sufficient to rule out the presence of Martian polymeric structures, obtaining additional data from polymer sequencing is recommended.

Detailed data analysis from sequencing would be used to determine if the sequences are characteristic of Earth life or are highly divergent. The differentiation of Martian life from terrestrial life may be clear due to vastly different chemical structures, different sequences of nucleic acids or nucleotide structures, and/or a chirality bias in amino acids and peptides. However, the possibility exists that life on Earth, Mars, or potentially throughout the solar system may stem from the same or very similar compounds and structures. This must be considered if any biological structures are detected.

Direct sequencing of polymerized nucleic acids or amino acids would be compared to terrestrial sequences in available public databases. An unmatched sequence may need to be treated as potentially hazardous until proven otherwise. If deemed to be non-hazardous (or from a terrestrial source), no further hazard testing may be warranted. However, a truly Martian signal would lead to an exciting and detailed research phase within containment. The difficulty in differentiation would also impact any future analysis, such as the use of amplification technology, particularly those incorporating canonical nucleotides such as PCR and Sanger sequencing. Because these technologies are likely to further complicate the conclusions of terrestrial vs Martian sequences, they were not considered useful within the scope of this protocol, but they may be considered for further investigation if biological hazards are detected.

A major challenge is that the biomass present in samples could be extremely low. Many of the current technologies available to detect and investigate potential life, such as cultivating a culture from a single cell (if present) or amplification of nucleic acids, potentially introduce terrestrial contamination that can give a false-positive result and rely on the idea that life on Mars would follow terrestrial hallmarks, specifically DNA (ATGC based) or ribonucleic acid (AUGC based) (2). Therefore, the SSAP-TT recommended the use of direct molecular sequencing, which may verify if the polymeric structures identified are unique from those in our terrestrial databases. The technology needed for molecular sequencing would need to detect and characterize small oligomers, non-canonical nucleic acids, single- and double-stranded nucleic acids, and canonical or non-canonical amino acids as potential indicators of life while also being capable of assessing their similarity to known terrestrial life. Technology also needs to be sensitive enough to detect extremely low quantities of these compounds in the presence of a potentially high-salt, highly oxidizing matrix. Currently, the development of high-throughput software with *de novo* or agnostic sequencing remains a mandatory process for identification without database searching, owing to the absence of reference sequences. Therefore, methods used for agnostic detection and sequencing of novel biological macromolecules are proposed (based on high-resolution sequencers and mass spectrometers (43, 44). It was also expected that such technology will continue to innovate.

A further consideration was extraction methods/efficiency of samples for sequencing. Nucleic acid extractions can provide information about living, dead, inactive, or dormant microbial populations. Analysis of nucleic acids from a natural low-biomass sample would be difficult due to extraction efficiency (45, 46). Low biomass requires caution with the subsampling to capture the sample with the highest quantity of potential nucleic acids, but also with consideration of the geochemistry of the sample and the presence of extraction inhibitors. For example, minerals can be natural extraction inhibitors since cells, viral structures, and nucleic acids may adsorb onto mineral matrices. Additionally, nucleic acids may bind to divalent and trivalent cations, and can be destroyed by iron redox cycling and acidic conditions. Therefore, extraction protocols need to be carefully defined (43, 47–49). Similar challenges and opportunities for research for the extraction and analysis of peptides/proteins are likely due to variability in their hydrophobicity and affinity to surfaces (50).

Based on previous low biomass studies in Martian analog environments, life-detection technology for assessing samples returned from Mars would likely need to meet detection requirements of 0.5 pg to 20 × 10^−10^ g of DNA per 1 g of rock (i.e., 0.00052 ppb) (43). Nanopore sequencing using the MinION technology could characterize species with as little as 2 pg of DNA with 50 active nanopores without the use of amplification. An advantage is that nanopore may be more amenable to different types of nucleic acids since the nucleic acid travels through a nanopore and identifies bases present by a change detection, while other platforms rely on a polymerase to incorporate fluorophore labeled nucleobases (43). Nanopore technology, along with improved base-calling algorithms and software application, shows promise in applications to sample return research, and further advancements in sensitivity and accuracy are expected (51). Another example utilized a multiplex fluorescence sandwich immunoassay to detect heterotrophic bacteria and cyanobacteria in Martian analog samples to detect evidence of life. This technology would also be an example requiring further development (52).

Similar advances are being made in polypeptide sequencing. Liquid chromatography-tandem mass spectrometry analysis has become the method of choice for the agnostic qualitative and quantitative characterization of relatively small, biologically active polypeptides, such as toxins and antibacterial agents, as well as protein mixtures isolated from all kinds of living organisms. There are two approaches for protein identification, top-down and bottom-up proteomics. In the bottom-up approach, peptides are first digested and then identified using MS analysis, whereas in the top-down approach, intact peptides and proteins are analyzed. This method has been used to identify small natural polypeptides such as venom toxins of less than 10 AA (<1 kDa) (53) and to detect bacterial and fungal toxins in different soil textures with a broad range of mineral and soil organic matter constituents, with concentrations as little as 0.5 ng of protein/g of soil (44).

## FUTURE PERSPECTIVES

Overall, the proposed SSAP assumes risk of biological hazards if modern Martian organic agents are detected in returned samples. In this case, a hold-and-review step is recommended, with a further program of biological safety containment and testing that should be undertaken. All samples should be held within containment (unless otherwise sterilized by an appropriate method) until testing has been completed and a statistical threshold indicating the absence of biological hazard has been attained. The protocol allows for this assessment by starting with prior knowledge and the cumulative analysis of data from the samples to an acceptable risk tolerance. The Bayesian statistical approach offers greater transparency and interpretability, which can be updated as new evidence becomes available. If significant questions remain with samples following analysis, a review team of subject matter experts should be assembled at that stage to determine the next steps.

Our discussions also highlighted areas of future research and development that could aid in the further implementation of this protocol. These included

- Knowledge of signal-to-noise ratio of biotic/abiotic components through simulations and experimentation from diverse or simulative environments.
- Optimizing extraction methods to assess potential abiotic components.
- Advanced data analysis and identification of limits of detection in low-biomass samples.
- Development of protocols for releasing samples out of high containment to include sterilization.
- Contamination knowledge (in the form of a catalog or database).
- Test instrument sensitivity and analytic methodology.
- Methods of extraction and fresh fracture surface mount preparation.

The concepts outlined in this report have the potential for significant impacts in other areas of environmental and applied microbiology. In addition to basic research into forms of life (both extinct and extant) in various Earth environments, the use of the detection of biosignatures and an overarching Bayesian statistical approach may be of use in industrial applications. This includes determining the cumulative statistical risk associated with the manufacturing of sterile products to innovate current requirements for traditional endpoint microbiological and/or chemical testing. Another is the application of biosignature detection as routine and rapid microbiological test methods to replace growth methods.

## References

[1] Ammann W, Barros J, Bennett A, Bridges J, Fragola J, Kerrest A, Marshall-Bowman K, Raoul H, Rettberg P, Rummel J, Salminen M, Stackebrandt E, Walter N. 2012. Mars sample return backward contamination – strategic advice and requirements—report from the ESF-ESSC Study group on MSR planetary protection requirements. European Science Foundation. https://elib.dlr.de/78092.

[2] Kminek G, Benardini JN, Brenker FE, Brooks T, Burton AS, Dhaniyala S, Dworkin JP, Fortman JL, Glamoclija M, Grady MM, et al.. 2022. COSPAR sample safety assessment framework (SSAF). Astrobiology 22:S–186. doi:10.1089/ast.2022.0017 35653292

[3] NASEM. 2019. An astrobiology strategy for the search for life in the universe. National Academies Press. https://nap.nationalacademies.org/catalog/25252/an-astrobiology-strategy-for-the-search-for-life-in-the-universe. 30986006

[4] NASEM. 2023. Origins, worlds, and life: a decadal strategy for planetary science and astrobiology 2023-2032. National Academies Press. 10.17226/26522.

[5] Teece BL, Beaty DW, Graham HV, McDonnell G, Sherwood Lollar B, Siljeström S, Steele A, Mackelprang R, SSAP Tiger Team. 2025. The abiotic background as a central component of a sample safety assessment protocol for sample return. Astrobiology 25:671–693. doi:10.1177/15311074251382156 41071731

[6] Goudge TA, Mustard JF, Head JW, Fassett CI, Wiseman SM. 2015. Assessing the mineralogy of the watershed and fan deposits of the Jezero crater paleolake system, Mars. JGR Planets 120:775–808. doi:10.1002/2014JE004782

[7] Mangold N, Gupta S, Gasnault O, Dromart G, Tarnas JD, Sholes SF, Horgan B, Quantin-Nataf C, Brown AJ, Le Mouélic S, et al.. 2021. Perseverance rover reveals an ancient delta-lake system and flood deposits at Jezero crater, Mars. Science 374:711–717. doi:10.1126/science.abl4051 34618548

[8] Stack KM, Williams NR, Calef F 3rd, Sun VZ, Williford KH, Farley KA, Eide S, Flannery D, Hughes C, Jacob SR, et al.. 2020. Photogeologic map of the perseverance rover field site in Jezero crater constructed by the Mars 2020 science team. Space Sci Rev 216:127. doi:10.1007/s11214-020-00739-x 33568875 PMC7116714

[9] Farley KA, Stack KM, Shuster DL, Horgan BHN, Hurowitz JA, Tarnas JD, Simon JI, Sun VZ, Scheller EL, Moore KR, et al.. 2022. Aqueously altered igneous rocks sampled on the floor of Jezero crater, Mars. Science 377. doi:10.1126/science.abo2196 36007009

[10] Bosak T, Shuster DL, Scheller EL, Siljeström S, Zawaski MJ, Mandon L, Simon JI, Weiss BP, Stack KM, Mansbach EN, et al.. 2024. Astrobiological potential of rocks acquired by the perseverance rover at a sedimentary fan front in Jezero Crater, Mars. AGU Advances 5:e2024AV001241. doi:10.1029/2024AV001241

[11] Zorzano MP, Martínez G, Polkko J, Tamppari LK, Newman C, Savijärvi H, Goreva Y, Viúdez-Moreiras D, Bertrand T, Smith M, et al.. 2024. Present-day thermal and water activity environment of the Mars Sample Return collection. Sci Rep 14:7175. doi:10.1038/s41598-024-57458-4 38532041 PMC10965995

[12] Westall F, Foucher F, Bost N, Bertrand M, Loizeau D, Vago JL, Kminek G, Gaboyer F, Campbell KA, Bréhéret JG, Gautret P, Cockell CS. 2015. Biosignatures on Mars: what, where, and how? implications for the search for Martian life. Astrobiology 15:998–1029. doi:10.1089/ast.2015.1374 26575218 PMC4653824

[13] NRC. 1997. Mars sample return: issues and recommendations. National Academies Press. 10.17226/5563.

[14] Farley KA, Williford KH, Stack KM, Bhartia R, Chen A, de la Torre M, Hand K, Goreva Y, Herd CDK, Hueso R, et al.. 2020. Mars 2020 mission overview. Space Sci Rev 216:142. doi:10.1007/s11214-020-00762-y

[15] Rummel JD, Beaty DW, Jones MA, Bakermans C, Barlow NG, Boston PJ, Chevrier VF, Clark BC, de Vera J-PP, Gough RV, Hallsworth JE, Head JW, Hipkin VJ, Kieft TL, McEwen AS, Mellon MT, Mikucki JA, Nicholson WL, Omelon CR, Peterson R, Roden EE, Sherwood Lollar B, Tanaka KL, Viola D, Wray JJ. 2014. A new analysis of Mars “special regions”: findings of the second MEPAG special regions science analysis group (SR-SAG2). Astrobiology 14:887–968. doi:10.1089/ast.2014.1227 25401393

[16] Rummel JD, Race MS, DeVincenzi DL, Schad PJ, Stabekis PD, Viso M, Acevedo SE. 2002. A draft test protocol for detecting possible biohazards in martian samples returned to Earth. Mars Sample Handling Protocol (MSHP) Workshop Series. https://ntrs.nasa.gov/citations/20030053046.

[17] Assurance M. 2021. NPR 8715.24. Planetary protection provisions for robotic extraterrestrial missions. Available from: https://nodis3.gsfc.nasa.gov/displayDir.cfm?t=NPR&c=8715

[18] Benardini N, Lalime EN. 2025. NASA planetary protection handbook. Available from: https://ntrs.nasa.gov/citations/20240016475

[19] NASEM. 2007. An astrobiology strategy for the exploration of Mars. National Academies Press. 10.17226/11937.

[20] Cooper M, Chen F, Guan L, Hinzer AA, Kazarians G, Ly C, Shirey TB, Stott K. 2023. Planetary protection implementation and verification approach for the Mars 2020 mission. Astrobiology 23:825–834. doi:10.1089/ast.2022.0046 37405744

[21] Sessions AL, Magnabosco C, Barton HA, Burkhardt C, Dworkin JP, Freissinet C, French KL, Glavin DP, Leys N, Maixner F, Olsson-Francis K, Probst AJ, Quitté G, Rampe E, Steele A, Carrier BL, Hays LE, Thiessen F, Paardekooper D, Hutzler A, Harrington AD, Teece BL. 2025. Planning considerations related to contamination control for the return and analysis of Martian samples. Astrobiology 25:694–724. doi:10.1177/15311074251382157 41043962

[22] Sephton MA, Steele A, Westall F, Schubotz F. 2025. Organic matter and biomarkers: Why are samples required? Proc Natl Acad Sci U S A 122:e2404256121. doi:10.1073/pnas.2404256121 39761399 PMC11745315

[23] Alleon J, Summons RE. 2019. Organic geochemical approaches to understanding early life. Free Radic Biol Med 140:103–112. doi:10.1016/j.freeradbiomed.2019.03.005 30858060

[24] McDonnell G, Hansen J. 2020. Block’s disinfection, sterilization, and preservation. 6th ed. Wolters Kluwer.

[25] Wenger LM, Davis CL, Isaksen GH. 2002. Multiple controls on petroleum biodegradation and impact on oil quality. SPE Reservoir Evaluation & Engineering 5:375–383. doi:10.2118/80168-PA

[26] Scalan ES, Smith JE. 1970. An improved measure of the odd-even predominance in the normal alkanes of sediment extracts and petroleum. Geochim Cosmochim Acta 34:611–620. doi:10.1016/0016-7037(70)90019-0

[27] Alexander R, Kagi RI, Rowland SJ, Sheppard PN, Chirila TV. 1985. The effects of thermal maturity on distributions of dimethylnaphthalenes and trimethylnaphthalenes in some Ancient sediments and petroleums. Geochim Cosmochim Acta 49:385–395. doi:10.1016/0016-7037(85)90031-6

[28] Martínez M, Escobar M. 1995. Effect of coal weathering on some geochemical parameters. Org Geochem 23:253–261. doi:10.1016/0146-6380(94)00115-H

[29] Matthewman R, Crawford IA, Jones AP, Joy KH, Sephton MA. 2016. Organic matter responses to radiation under lunar conditions. Astrobiology 16:900–912. doi:10.1089/ast.2015.1442 27870583 PMC5273402

[30] Montgomery W, Bromiley GD, Sephton MA. 2016. The nature of organic records in impact excavated rocks on Mars. Sci Rep 6:30947. doi:10.1038/srep30947 27492071 PMC4974657

[31] Allton JH, Hittle JD, Mickelson ET, Stansbery EK. 2016. Cleaning genesis sample return canister for flight: lessons for planetary sample return. Available from: https://ntrs.nasa.gov/citations/20160002409

[32] Summons RE, Sessions AL, Allwood AC, Barton HA, Beaty DW, Blakkolb B, Canham J, Clark BC, Dworkin JP, Lin Y, Mathies R, Milkovich SM, Steele A. 2014. Planning considerations related to the organic contamination of martian samples and implications for the Mars 2020 rover. Astrobiology 14:969–1027. doi:10.1089/ast.2014.1244 25495496

[33] Eigenbrode JL, Summons RE, Steele A, Freissinet C, Millan M, Navarro-González R, Sutter B, McAdam AC, Franz HB, Glavin DP, Archer PD Jr, Mahaffy PR, Conrad PG, Hurowitz JA, Grotzinger JP, Gupta S, Ming DW, Sumner DY, Szopa C, Malespin C, Buch A, Coll P. 2018. Organic matter preserved in 3-billion-year-old mudstones at Gale crater, Mars. Science 360:1096–1101. doi:10.1126/science.aas9185 29880683

[34] Grady MM, Verchovsky AB, Wright IP. 2004. Magmatic carbon in Martian meteorites: attempts to constrain the carbon cycle on Mars. Int J Astrobiology 3:117–124. doi:10.1017/S1473550404002071

[35] Scheller EL, Bosak T, McCubbin FM, Williford K, Siljeström S, Jakubek RS, Eckley SA, Morris RV, Bykov SV, Kizovski T, et al.. 2024. Inorganic interpretation of luminescent materials encountered by the perseverance rover on Mars. Sci Adv 10:eadm8241. doi:10.1126/sciadv.adm8241 39321302 PMC11423895

[36] Sharma S, Roppel RD, Murphy AE, Beegle LW, Bhartia R, Steele A, Hollis JR, Siljeström S, McCubbin FM, Asher SA, et al.. 2023. Diverse organic-mineral associations in Jezero crater, Mars. Nature 619:724–732. doi:10.1038/s41586-023-06143-z 37438522 PMC10371864

[37] Steele A, McCubbin FM, Fries M, Kater L, Boctor NZ, Fogel ML, Conrad PG, Glamoclija M, Spencer M, Morrow AL, Hammond MR, Zare RN, Vicenzi EP, Siljeström S, Bowden R, Herd CDK, Mysen BO, Shirey SB, Amundsen HEF, Treiman AH, Bullock ES, Jull AJT. 2012. A reduced organic carbon component in martian basalts. Science 337:212–215. doi:10.1126/science.1220715 22628557

[38] Stern JC, Malespin CA, Eigenbrode JL, Webster CR, Flesch G, Franz HB, Graham HV, House CH, Sutter B, Archer PD Jr, Hofmann AE, McAdam AC, Ming DW, Navarro-Gonzalez R, Steele A, Freissinet C, Mahaffy PR. 2022. Organic carbon concentrations in 3.5-billion-year-old lacustrine mudstones of Mars. Proc Natl Acad Sci USA 119:e2201139119. doi:10.1073/pnas.2201139119 35759667 PMC9271195

[39] Maltais TR, Boeder P, Soares C, Mennella J, Heinz N, Gomez V, Alred J, Anderson MS, Clark I. 2023. An accounting of contamination control requirements, implementation, and verification of the sample tubes for the Mars 2020 mission and future return sample science. Astrobiology 23:846–861. doi:10.1089/ast.2022.0049 37192487

[40] Chen F, Ly C, Mikellides I, Bernard D, Cooper M. 2023. Mars 2020 mission biological return sample contamination control approach and verification. Astrobiology 23:862–879. doi:10.1089/ast.2022.0048 37584747

[41] Schubert WW, Seto EP, Hinzer AA, Guan L. 2023. Identification and archive of Mars 2020 spacecraft microbial isolates. Astrobiology 23:835–845. doi:10.1089/ast.2022.0052 37584746

[42] Green SJ, Torok T, Allen JE, Eloe-Fadrosh E, Jackson SA, Jiang SC, Levine SS, Levy S, Schriml LM, Thomas WK, Wood JM, Tighe SW. 2023. Metagenomic methods for addressing NASA’s planetary protection policy requirements on future missions: a workshop report. Astrobiology 23:897–907. doi:10.1089/ast.2022.0044 37102710 PMC10457625

[43] Basapathi Raghavendra J, Zorzano MP, Kumaresan D, Martin-Torres J. 2023. DNA sequencing at the picogram level to investigate life on Mars and Earth. Sci Rep 13:15277. doi:10.1038/s41598-023-42170-6 37714862 PMC10504319

[44] Kappenberg A, Juraschek LM. 2021. Development of a LC-MS/MS method for the simultaneous determination of the mycotoxins deoxynivalenol (DON) and zearalenone (ZEA) in soil matrix. Toxins (Basel) 13:470. doi:10.3390/toxins13070470 34357942 PMC8310301

[45] Direito SOL, Marees A, Röling WFM. 2012. Sensitive life detection strategies for low-biomass environments: optimizing extraction of nucleic acids adsorbing to terrestrial and Mars analogue minerals. FEMS Microbiol Ecol 81:111–123. doi:10.1111/j.1574-6941.2012.01325.x 22329626

[46] Mojarro A, Ruvkun G, Zuber MT, Carr CE. 2017. Nucleic acid extraction from synthetic mars analog soils for in situ life detection. Astrobiology 17:747–760. doi:10.1089/ast.2016.1535 28704064 PMC5567878

[47] Bashir AK, Wink L, Duller S, Schwendner P, Cockell C, Rettberg P, Mahnert A, Beblo-Vranesevic K, Bohmeier M, Rabbow E, et al.. 2021. Taxonomic and functional analyses of intact microbial communities thriving in extreme, astrobiology-relevant, anoxic sites. Microbiome 9:50. doi:10.1186/s40168-020-00989-5 33602336 PMC7893877

[48] Fortunato CS, Huber JA. 2016. Coupled RNA-SIP and metatranscriptomics of active chemolithoautotrophic communities at a deep-sea hydrothermal vent. ISME J 10:1925–1938. doi:10.1038/ismej.2015.258 26872039 PMC5029171

[49] Ugwuanyi IR, Fogel ML, Bowden R, Steele A, De Natale G, Troise C, Somma R, Piochi M, Mormone A, Glamoclija M. 2023. Comparative metagenomics at Solfatara and Pisciarelli hydrothermal systems in Italy reveal that ecological differences across substrates are not ubiquitous. Front Microbiol 14:1066406. doi:10.3389/fmicb.2023.1066406 36819055 PMC9930910

[50] Keiblinger KM, Wilhartitz IC, Schneider T, Roschitzki B, Schmid E, Eberl L, Riedel K, Zechmeister-Boltenstern S. 2012. Soil metaproteomics - comparative evaluation of protein extraction protocols. Soil Biol Biochem 54:14–24. doi:10.1016/j.soilbio.2012.05.014 23125465 PMC3413887

[51] Chen P, Sun Z, Wang J, Liu X, Bai Y, Chen J, Liu A, Qiao F, Chen Y, Yuan C, Sha J, Zhang J, Xu LQ, Li J. 2023. Portable nanopore-sequencing technology: trends in development and applications. Front Microbiol 14:1043967. doi:10.3389/fmicb.2023.1043967 36819021 PMC9929578

[52] Azua-Bustos A, Fairén AG, González-Silva C, Prieto-Ballesteros O, Carrizo D, Sánchez-García L, Parro V, Fernández-Martínez MÁ, Escudero C, Muñoz-Iglesias V, et al.. 2023. Dark microbiome and extremely low organics in Atacama fossil delta unveil Mars life detection limits. Nat Commun 14:808. doi:10.1038/s41467-023-36172-1 36810853 PMC9944251

[53] Siemerink M, Schebb NH, Liesener A, Perchuc AM, Schöni R, Wilmer M, Hayen H, Karst U, Vogel M. 2010. Development of a fast liquid chromatography/mass spectrometry screening method for angiotensin‐converting enzyme (ACE) inhibitors in complex natural mixtures like snake venom. Rapid Comm Mass Spectrometry 24:687–697. doi:10.1002/rcm.4428 20162537

